# Author Correction: Microenvironment-responsive immunoregulatory electrospun fibers for promoting nerve function recovery

**DOI:** 10.1038/s41467-021-23438-9

**Published:** 2021-05-11

**Authors:** Kun Xi, Yong Gu, Jincheng Tang, Hao Chen, Yun Xu, Liang Wu, Feng Cai, Lianfu Deng, Huilin Yang, Qin Shi, Wenguo Cui, Liang Chen

**Affiliations:** 1 Department of Orthopedics, the First Affiliated Hospital of Soochow University, Orthopedic Institute, Soochow University, 188 Shizi Road, 215006 Suzhou, Jiangsu P. R. China; 2 Shanghai Key Laboratory for Prevention and Treatment of Bone and Joint Diseases, Shanghai Institute of Traumatology and Orthopaedics, Ruijin Hospital, Shanghai Jiao Tong University School of Medicine, 197 Ruijin 2nd Road, 200025 Shanghai, P. R. China; 3 Shanghai Public Health Clinical Center, Fudan University, 2901 Caolang Road, 200025 Shanghai, P. R. China; 4 Key Laboratory of Stem Cells and Biomedical Materials of Jiangsu Province and Chinese Ministry of Science and Technology, 199 Renai Road, 215123 Suzhou, P. R. China

**Keywords:** Drug delivery, Spinal cord injury, Biomaterials

Correction to: *Nature Communications* 10.1038/s41467-020-18265-3, published online 9 September 2020.

The original version of this Article contained an error in Fig. 3, in which the incorrect images were used in Fig. 3a for the ratio of 1:2. This has been corrected in both the PDF and HTML versions of the Article.

