# Supplementary material for: Microenvironment-responsive immunoregulatory electrospun fibers for promoting nerve function recovery
Source: Nat Commun. 2020 Sep 9;11:4504. doi: 10.1038/s41467-020-18265-3 (PMC7481196; doi:10.1038/s41467-020-18265-3)
Supplement: Supplementary file 1 — Supplementary Information [file 41467_2020_18265_MOESM1_ESM.pdf]

Supplementary Information

Microenvironment-Responsive Immunoregulatory Electrospun Fibers for Promoting Nerve Function Recovery

Xi et al,

Supplementary Methods

Characterization of fiber scaffold morphology. In the process of configuring microsol electrospinning solution, the particle size distribution of HA microsol particles in dichloromethane was measured by dynamic light scattering particle size analyzer (DLS). Prior to the observation, the samples were fixed to the sample table with conductive tape and sprayed with gold (SC7620, Quorum Technologies, UK). The fiber scaffold was observed by scanning electron microscope (SEM, S-4800, Hitachi, Japan) with the acceleration voltage of 10kV and magnification of 20000 times. A total of 100 fibers were randomly selected from each sample, and the fiber diameter was plotted and analyzed by ImageJ software. To characterize the orientation of the fiber scaffold, we randomly selected three SEM images from each group, and the overall direction of the fiber membrane was set to 0°. Orientation analysis through the orientation plugin of ImageJ software. The internal structure of a single fiber is verified by TEM at 120kV voltage. X-ray photoelectron spectroscopy (XPS, 250Xi, Thermo Scientific Escalab, USA) was used to analyze the changes of surface elements after the grafting of liposomes with spinning scaffolds. The topological morphology of the fiber surface was observed by atomic mechanics microscope (AFM, Bruker, Germany), and the average roughness of the fiber surface (Ra) was evaluated by NanoScope Analysis 1.7 software. In the above morphology characterization, the traditional electrospun fiber scaffold (aP) and the microsol electrospun fiber scaffold (MSaP) were introduced as the control.

Functional characterization of fiber scaffold. The acid-sensitive chemical bond Schiff base formed by the electrospinning of aldehyde cationic liposomes and amino polylactic acid (PLA) and the different functional groups carried by each scaffold were characterized by infrared spectroscopy (FTIR, Nicolet6700, Thermo scientific, USA). The flakes were prepared by potassium bromide grinding and scanned for 128 times with a resolution of 4cm^-1 and a range of 500~4000cm^-1.

The composite fiber scaffold was immersed in a solution containing 0.5% Triton X-100 to dissolve phospholipids, and as mentioned above, the amount of liposome-loaded pDNA fixed to the fiber scaffold was detected by dsDNA kit (Invitrogen, USA).

Compared with the original pDNA concentration and liposome encapsulation efficiency, the loading rate of pDNA graft on the fiber scaffold was calculated.

In order to study the mechanical properties of nerve scaffold support, 15.0×3.0×0.1mm samples were prepared by mould and tested in general mechanical test system (Hengyi, Shanghai, China) with a tensile speed of 10mm min^-1. The Young's modulus was obtained by calculating the slope of the stress-strain curve of the origin.

The degradation property was tested by immersing different fiber scaffolds in centrifugal tubes containing 30ml PBS at room temperature which was replaced every day. At each set time point, the fiber scaffold was lyophilized and weighed to calculate the degradation rate at each time point based on the initial mass as well as the residual mass.

To characterize the cytokine content in the fibers, three prepared MSaP-aL/p scaffolds were placed in three 50ml centrifuge tubes containing 2g DCM, centrifuged at 25155xg for 5 minutes, and the aPLA-containing supernatant was removed. This was repeated three times to ensure that all NGF in the organic phase precipitation. After adding 2ml PBS for resuspension, the ELISA kit was used to detect the NGF content, and the drug loading efficiency of electrospun fibers was calculated as follows (Eq. 1):

$$LE\ (\%) = \frac{M\ (\text{total drug in fibers})}{M\ (\text{total drug before spinning})} \qquad (1)$$

The static water contact angle on the surface of fiber scaffold was measured by contact angle meter (WCA, DSA25S, Data Physics Corporation) to evaluate the hydrophobicity of fiber scaffold.

The degree of porosity of each group scaffolds was determined by the pycnometer method [1]. Briefly, the weight of the pycnometer filled with ethanol was measured and labeled as W_1; the sample with a weight of Ws, and was immersed in ethanol. Subsequently, the sample was saturated by ethanol; additional ethanol was added to complete the volume of the pycnometer. Then, the pycnometer was weighted and labeled as W_2; the sample filled with ethanol was taken out of the pycnometer and the

residual weight of the ethanol and the pycnometer was labeled as W_3. The porosity of the membrane was calculated according to Equation (2):

$$\mathcal{E} = \frac{W_2 - W_3 - W_S}{W_1 - W_3} \times 100\% \qquad (2)$$

The porosity of each scaffold was obtained as the mean value of the porosity determined in three samples

Evaluation of cell biological characteristics. The survival rate of BMSCs on different fiber membranes was evaluated by living death staining kit (Invitrogen, Eugene, OR) staining after cultured for three days. The stained cells were observed and photographed under fluorescence microscope, with the fluorescence semi-quantitative analysis carried out using ImageJ software. Proliferation rate of BMSCs on membranes were investigated using cell counting kit-8 (CCK-8, Dojindo, Japan) at 1, 3 and 5 days after implantation. After incubation in working medium for 4 hours, 100ul working medium was absorbed into the 96-well plate, and measured by enzyme labeling instrument (BioTek, USA), at the wavelength of 450nm. All tests were repeated three times.

The adhesion of cells to fiber membranes was evaluated by immunofluorescence. After co-culture for one day, the cells were fixed with 4% paraformaldehyde, with the cell membrane perforated using the solution containing 0.5% Triton X-100. In order to avoid non-specific staining, BSA was used to block the cells at 4℃ overnight. The cells were washed with PBS three times before incubated with the first antibody Integrinβ1 (Novus, NBP2-36561, 1:200) at 4℃ overnight. The goat anti-mouse second antibody (Jackson, 115-545-003, 1:300) was incubated with cell at room temperature for 1h after washed for three times. Finally, the cytoskeleton and nucleus were stained with phalloidin and DAPI. The cover slides were removed from the culture plate, with cells observed and photographed under the fluorescence microscope, and the fluorescence semi-quantitative analysis was carried out by using ImageJ software. For the observation under scanning electron microscope, membranes seeded with cells

cultured for three days were rinsed with PBS for three times and fixed with paraformaldehyde for 30 min. After that, the ethanol gradient dehydration with volume ratio of 10%, 20%, 35%, 50%, 70%, 85%, 100% was performed, and the morphology of BMSCs on different fiber membranes was observed by SEM after critical point drying and surface gold spray treatment.

Characterization of inflammatory cytokines in BMM. Under the influence of responsive fiber membrane, the biological characteristics of BMMs cells changed on the level of gene expression after polarization, so the BMMs culture medium was collected at the same time point, and the pro-inflammatory cytokines IL-1β, TNF-α and anti-inflammatory related cytokines IL-10, TGF-β were evaluated respectively, using the ELISA kit (Multi Sciences, China), with the colorimetric changes were carried out with enzyme labeling instrument. On the 7th day of co-culture, the specific phenotypes of BMM F4/80 (Abcam, ab100790, 1:100) and the characteristic phenotypes of M1 and M2 macrophages iNOS (Abcam, ab49999, 1:100) and CD206 (Santa Cruz, sc-376108, 1:100), followed with goat anti-rabbit (Abcam, ab155079, 1:200) and goat anti-mouse (Abcam, ab150113, 1:400) second antibody incubated， and semi-quantitative using ImageJ software.

Characterization of neural differentiation. In order to evaluate the effect of fiber membrane on promoting neural differentiation of stem cells, BMSCs cultured on fiber membrane on the 10th day was stained with neuron specific markers. Specifically, after removing the culture medium and washing with PBS preheated to 37℃ for 3 times and 5 minutes each time, cells were fixed at room temperature with 4% paraformaldehyde for 30 minutes. After adding 0.3% Triton X-100 to perforate for 30min, the cell was washed with PBS three times, and then incubated with 5% BSA solution overnight. After the removal of BSA solution, the first antibody (rabbit anti-Tuj-1, Abcam, ab18207, 1:500) was added and incubated at 4℃ overnight. After 3 times of PBS washing, the samples were incubated with the second antibody (goat anti-rabbit, Abcam, ab150081, 1:400) at room temperature for 1h. The cytoskeleton and nucleus were

stained by phalloidin and DAPI. The staining results were observed and photographed by fluorescence microscope, and the fluorescence semi-quantitative analysis was carried out in ImageJ software. NSE (rabbit anti-NSE, Abcam, ab53025, 1:200), Tau protein (mouse anti-Tau, Abcam, ab80579, 1:250) and NF-200 (mouse anti-NF-200, Abcam, ab82259, 1:200) staining were performed by the same method mentioned above. Goat anti-mouse second antibody (alexa488, Abcam, ab150113, 1:400) was used for immunofluorescence staining of Tau protein and NF-200. Rat bone marrow mesenchymal stem cells (BMSCs) were also seeded into 24-well plates (n=3). When the cells were about 70% -80% fused, added MSaP-aL/p release solution (day 30 to 40, pH5.8). Five days later, the neuronal marker Tuj-1 was used to label differentiated neuron-like cells and semi-quantitative fluorescence analysis was performed by imageJ software to evaluate the long-term biological activity of NGF released from the fibrous membrane (r-NGF group). Normally cultured bone marrow mesenchymal stem cells were used as the negative control group (NC), and rat β-NGF cytokines were added as the positive control group (PC). Three separate dissociations were carried out to achieve biological triplicate (n=3).

QRT-PCR technique was used to evaluate the expression levels of neuron specific markers Tuj-1, NSE, Tau protein and NF-200 after stem cell differentiation. Glyceraldehyde-3-phosphate dehydrogenase (GAPDH) was used as the internal reference gene. Polymerase chain reaction amplification primers were designed by Genewiz and shown in Supplementary Table 5.

Characterization of foreign body reaction in vivo. Different fiber bundles were cut to the size of 10mm×10mm×0.1mm and implanted under the epidermis on the back of SD rats to evaluate the foreign body reaction in vivo. The specimens were harvested 2 weeks after operation. After fixed in 10% formalin solution, the specimens were sliced and stained with H&E to observe the inflammatory response zone, and randomly selected three fields of view for semi-quantitative and statistical analysis of inflammation area using ImageJ software (n=3).

Fluorescently labeled immune factors. After the animal's chest was fully exposed, 100ml saline followed by 100ml 4% paraformaldehyde were injected into the left ventricle respectively, after which the spinal cord specimens were removed and soaked in 4% paraformaldehyde for 24 hours, and then embedded in paraffin. The specimens were sliced along the long axis of the spinal cord with the thickness of 5μm. After dewaxing with xylene and ethanol, 0.3% hydrogen peroxide was used to block the activity of endogenous peroxidase, and then the specimen was incubated with primary antibody against IL-4 (mouse anti-IL-4, Santa Cruz, sc-53082, 1:100), TNF-α (rabbit anti-TNF-α, Abcam, ab6671, 1:500) and IL-10 (rabbit anti-IL-10, Abcam, ab9969, 1:300) at 4℃ overnight. After washing with PBS for three times, the second antibody including goat anti-mouse (Cy3, Service bio, GB21301, 1:500), goat anti-rabbit (HRP, Service bio, GB23303, 1:400) , goat anti-rabbit (Cy3, Service bio, GB21303, 1:400) was added followed by incubation for 1h. Photographing by fluorescence microscope, and semi-quantitative analysis of five randomly selected images which had been corrected for optical density, de-background, and finally normalized the staining intensity by the number of cell nuclei using ImageJ software.

Assessment of inflammatory genes in injured local tissues. To evaluate the transfection performance and immunoregulatory function after fiber bundles implantation, three randomly selected spinal cord specimens were dehydrated in ethanol followed by xylene (n=3). Then total RNA of spinal cord was isolated by using the RNAprep Pure FFPE kit (TIANGEN BIOTECH, China) according to the manufacturer's instructions. cDNA was synthesized with 1μg of RNA using a Hiscript II 1st Strand cDNA Synthesis kit (Vazyme, Nanjing, China). Quantitative RT-PCR was carried out in a 20μL total volume ChamQ Universal SYBR qPCR Master Mix (Vazyme, Nanjing, China). The gene expression levels were normalized to GAPDH. Primers were shown in Supplementary Table 6.

Quantitative serum immune factors. The local severe inflammatory reaction of spinal cord injury produces a large number of immune factors into the systemic circulation with the blood. We used the way of clinical detection of inflammatory factors to initially evaluate the changes of inflammatory response after fiber bundles implantation. Briefly, three animals were randomly selected from each group, and the blood was collected with a centrifuge tube containing coagulant during cardiac perfusion (n=3). The blood was placed at room temperature for 30 minutes and then centrifuged at 1006.2xg for 5 minutes. The upper serum was collected and detected by ELISA kit (Multi Sciences, China).

IL-4 plasmid transfection and macrophage phenotype. Spinal cord specimens were collected 7 days after operation for frozen sections following procedure reported previously (MSaP-aL/p group was set as the experimental group, the unloaded plasmid group (MSaP-aL) and the eGFP plasmid loaded group (MSaP-aL/g) were used as the control group) [2]. Briefly, after intraperitoneal anesthesia, 4% paraformaldehyde and saline were perfused into the left ventricle of rat respectively, and the spinal cord specimens were quickly collected and immersed in 4% paraformaldehyde overnight. After subsequently immersed in 30% sucrose cryoprotectant overnight, the spinal cord samples were frozen for section with a longitudinal thickness of 15μm for following immunofluorescent staining with CD206 antibody (Abcam, ab195192, 1:100) coupled with co-staining of nucleus with DAPI.

Pharmacokinetics of NGF in spinal cord. Spinal cord specimens centered on the injury site were collected on 1, 6, 11, 16, 21 days after operation. After weighing, specimens were immersed in PBS buffer containing 1% PMSF with 5 times mass volume of sample, and cut into small pieces. The tissue homogenate was prepared and centrifuged at 12000g for 10 minutes to obtain supernatant. The NGF level in spinal cord was then determined by ELISA (Solarbio Life Sciences, SEKR-0015) with a standard curve established as manuscript's instruction.

Supplementary Figures:

a

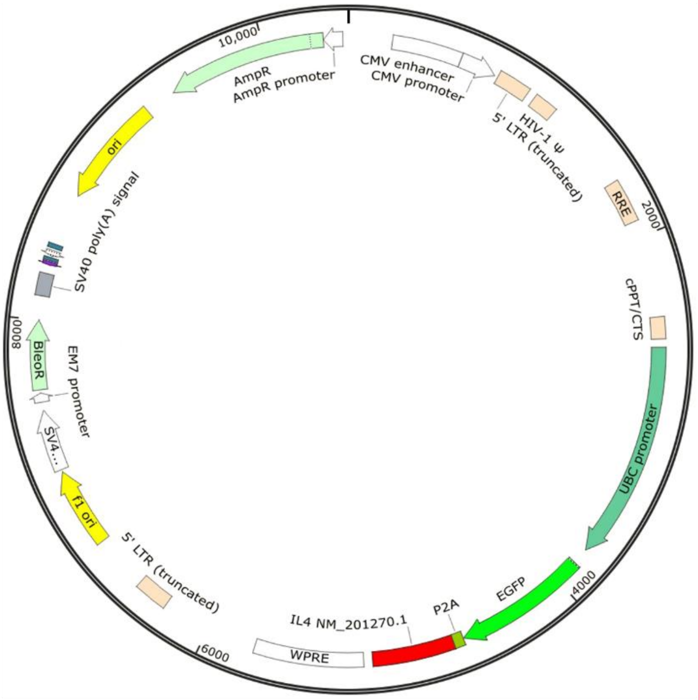

b

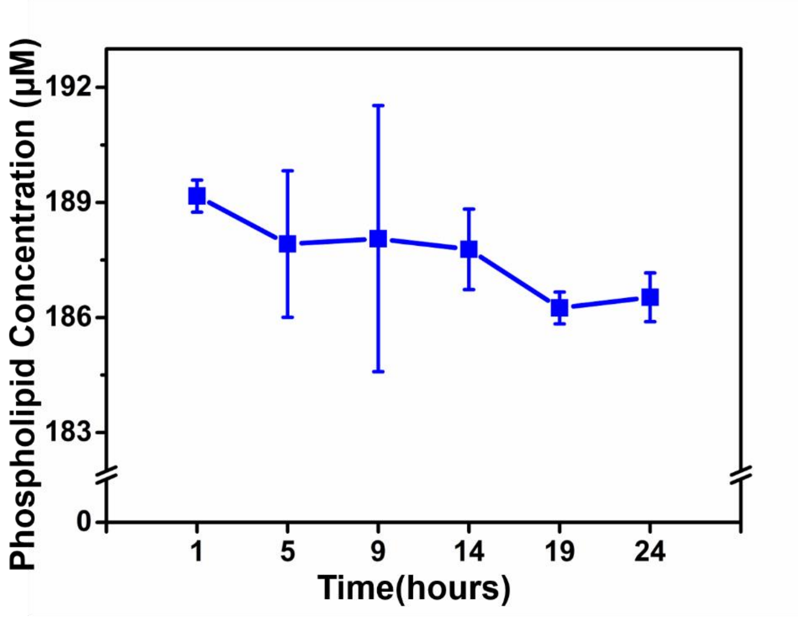

Supplementary Figure 1. Plasmid profile and stability of liposomes. a IL-4-eGFP plasmid profile. EGFP was designed for the report protein of IL-4 expression and **b** Detection of stability of aldehyde cationic liposomes. (n=3, all values were mean ± std. dev)

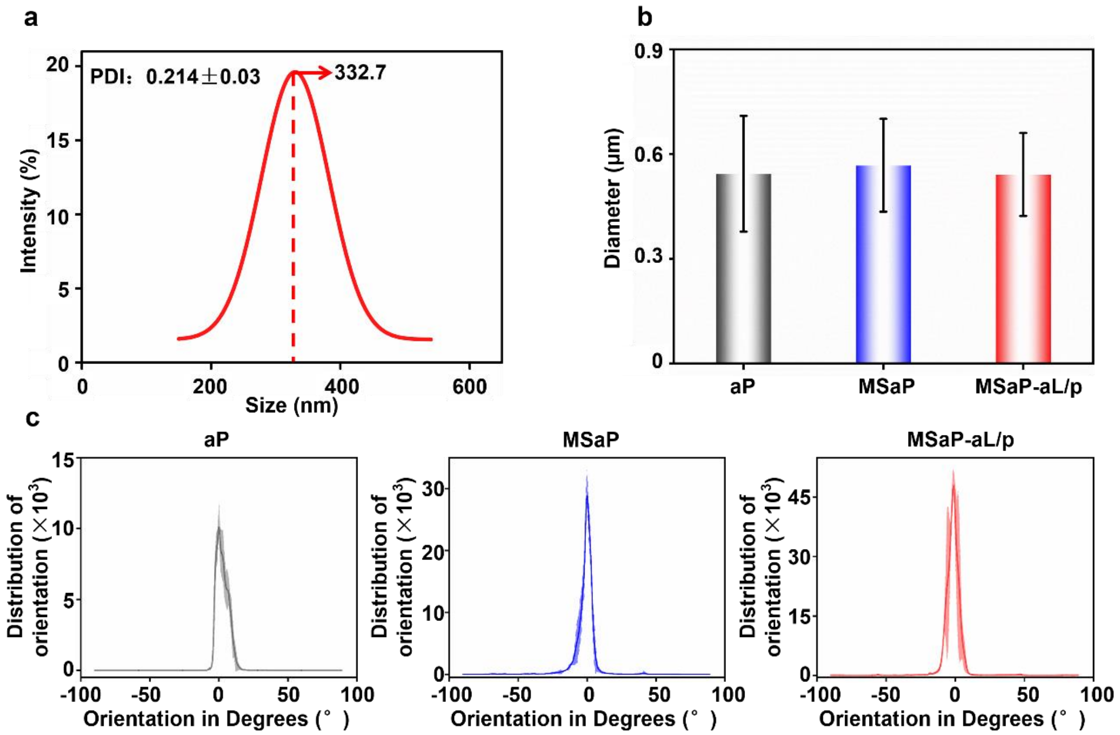

Supplementary Figure 2. Characterization of fiber scaffold morphology. a Evaluation of particle size distribution and PDI of HA coated with β-NGF (n=3, PDI values were mean ± std. dev and p values were determined by one-way analysis of variance (ANOVA) with a Tukey's post-hoc test). **b** Comparison of diameter distribution of different fiber scaffolds (n=100, diameter distribution values were mean ± std. dev and p values were determined by one-way analysis of variance (ANOVA) with a Tukey's post-hoc test). **c** Analysis of orientation of different fiber scaffolds. (n=3, orientation values were mean ± std. dev)

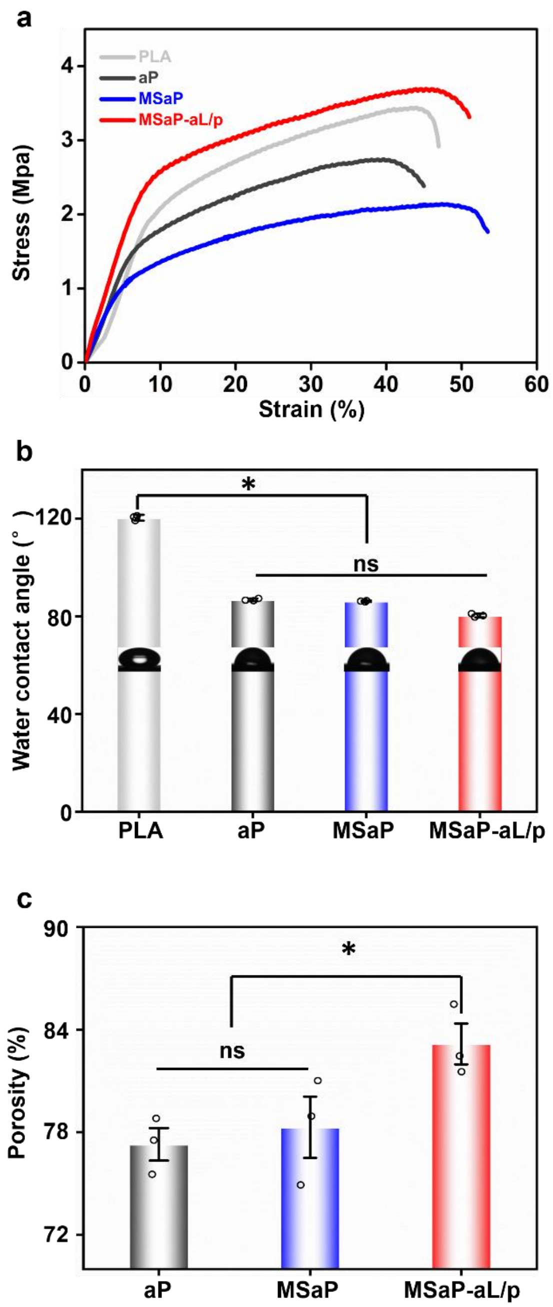

Supplementary Figure 3. Characterization of different fiber scaffolds. a Stress-strain curve of nanofibrous scaffolds. **b** Evaluation of water contact angle of different fiber scaffolds (n=3, water contact angle values were mean ± std. dev., $^{*}p < 0.05$ when comparing PLA group and three other groups via one-way analysis of variance (ANOVA) with a Tukey's post-hoc test., ns, not significant). **c** Porosity of different fiber scaffolds (n=3, porosity values were mean ± std. dev and $^{*}p<0.05$ when comparing MSaP-aL/p and two other groups via one-way analysis of variance (ANOVA) with a Tukey's post-hoc test).

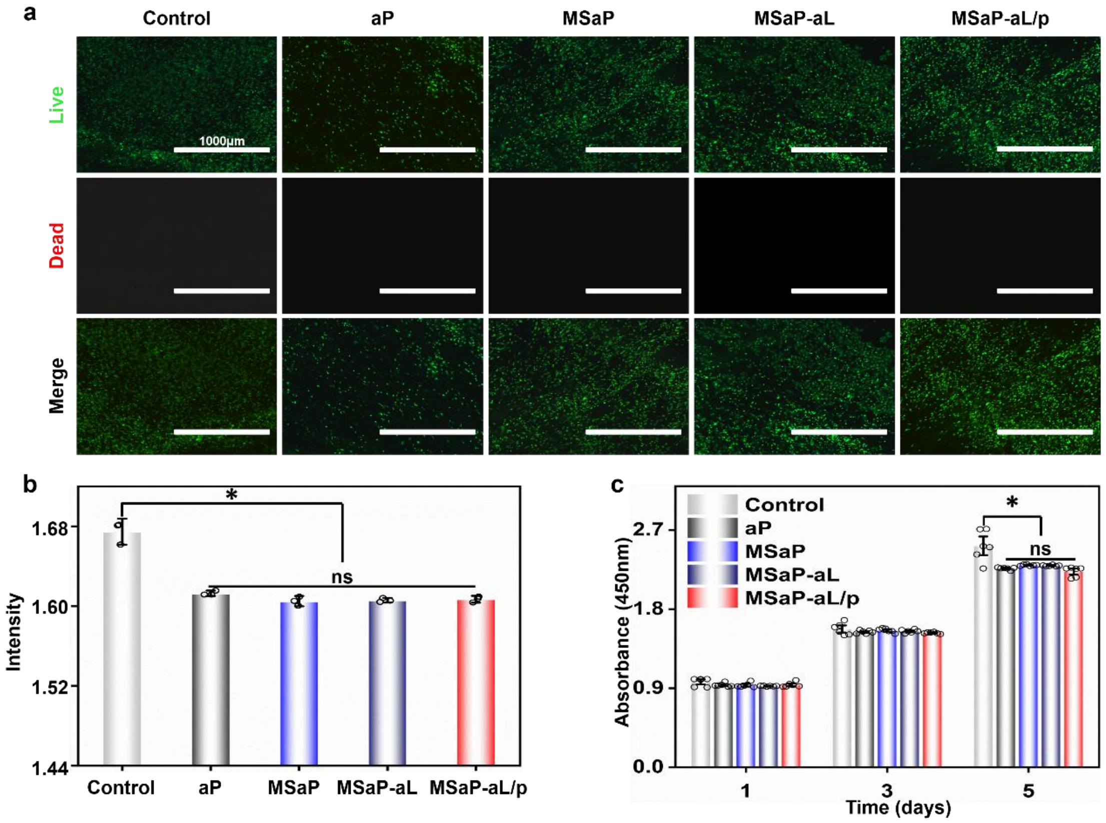

Supplementary Figure 4. Cell survival and proliferation assays. a Live/dead (green/red) fluorescence staining images (scale bars were 1000μm, 3 independent experiments). **b** Fluorescence semi-quantitative analysis of living cells (n=3, fluorescence density values were mean ± std. dev., $^{*}p < 0.05$ when comparing control group and four other groups via one-way analysis of variance (ANOVA) with a Tukey's post-hoc test., ns, not significant). **c** Detection of cell proliferation by CCK8 kit at 1, 3 and 5 days (n=6, absorbance values were mean ± std. dev., $^{*}p < 0.05$ when comparing control group and four other groups via two-way analysis of variance (ANOVA) with a Tukey's post-hoc test., ns, not significant).

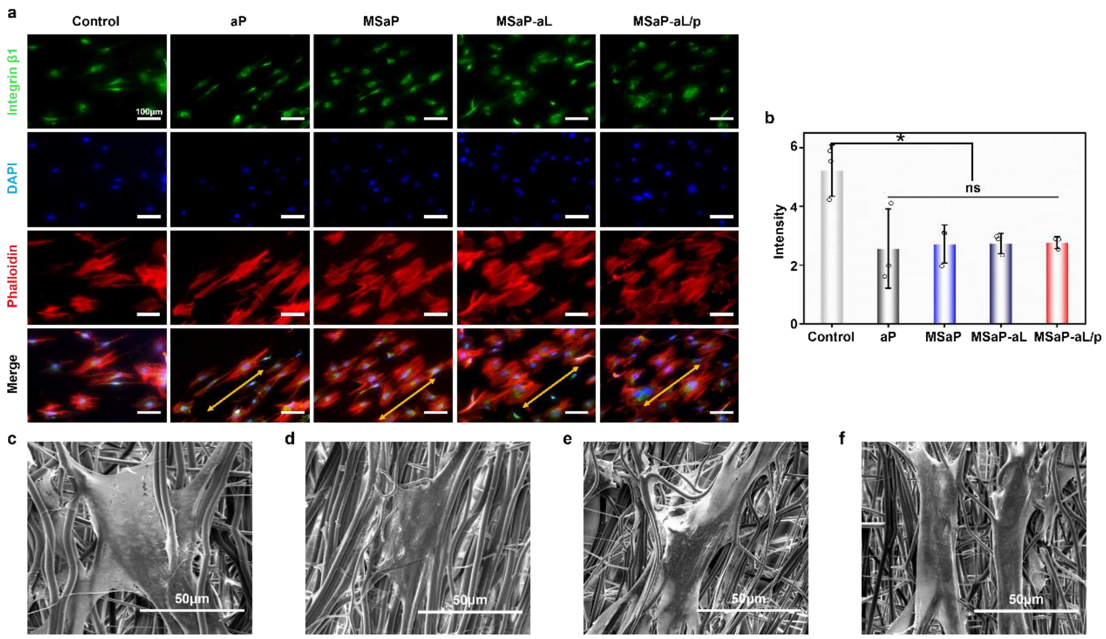

Supplementary Figure 5. Cell adhesion assays of scaffolds. a Integrin β1 immunofluorescence staining images of BMSCs inoculated with fiber scaffolds for 1 day (scale bar were 100μm, 3 independent experiments). **b** Fluorescence semi-quantitative analysis of Integrin β1(n=3, integrin β1 intensity values were mean ± std. dev., $^{*}p < 0.05$ when comparing control group and four other groups via one-way analysis of variance (ANOVA) with a Tukey's post-hoc test., ns, not significant). **c, d, e, f** SEM images of BMSCs cultured on different fiber scaffolds for three days, scale bars were 50μm, 4 independent experiments).

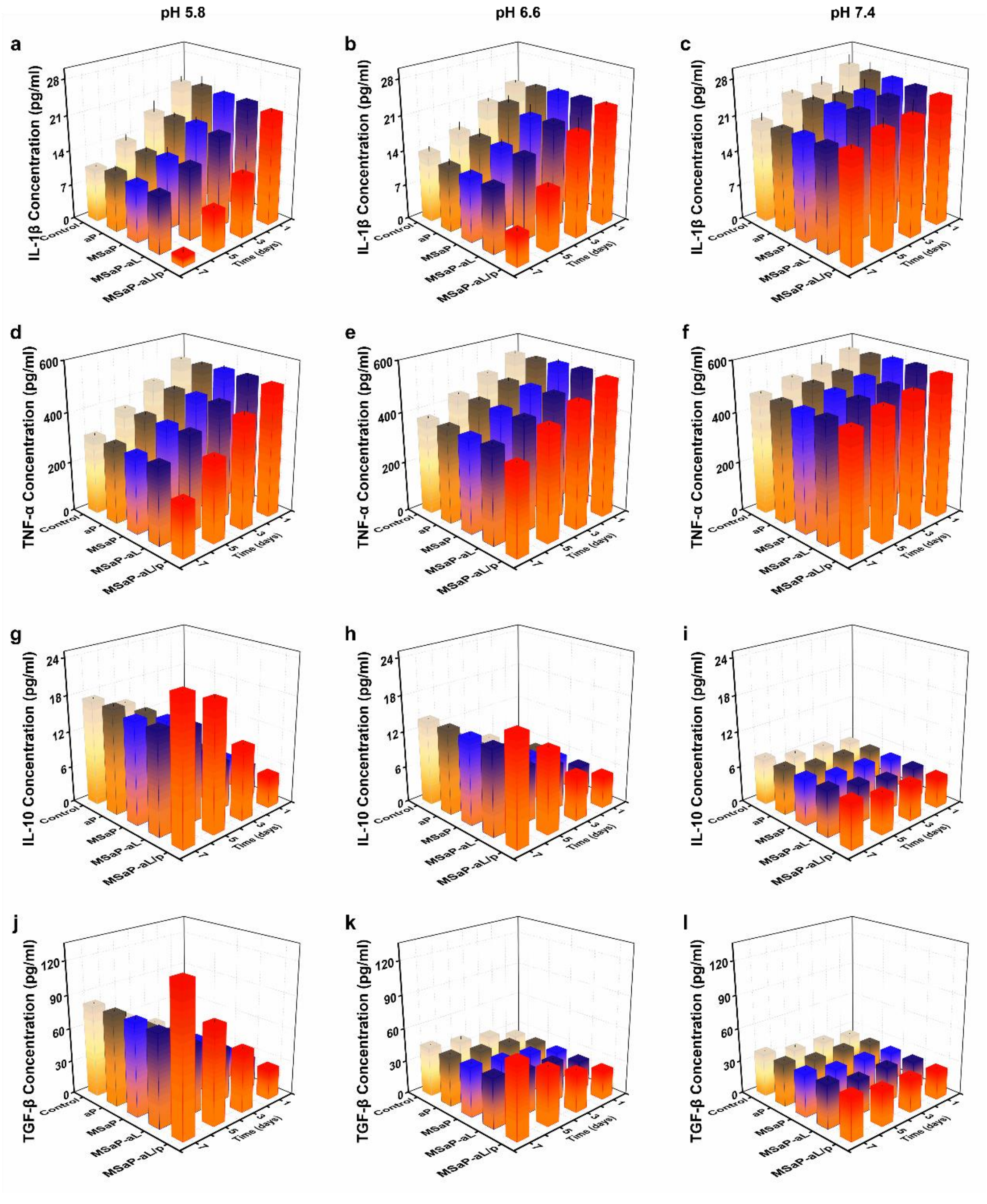

Supplementary Figure 6. Factors secreted by BMM at different pHs by ELISA. a, b, c BMM secreted pro-inflammatory factor IL-1β at different pHs (n=3, values were mean ± std. dev., p<0.001 when comparing MSaP-aL/p and other four groups (control, aP, MSaP and MSaP-aL) on the 3rd, 5th and 7th day at pH5.8 condition, and p < 0.001 when comparing compared MSaP-aL/p and other four groups on the 5th and 7th day at pH6.6 condition via two-way analysis of variance (ANOVA) with a Tukey's post-hoc test). **d, e, f** BMM secreted pro-inflammatory factor TNF-α at different pHs (n=3, values

were mean ± std. dev., $p<0.0001$ when comparing MSaP-aL/p and other four groups on the 3rd and 7th day at pH5.8 condition, $p<0.001$ when comparing MSaP-aL/p and other four groups on the 5th day at pH5.8 condition, and $p<0.001$ when comparing MSaP-aL/p and other four groups on the 7th day at pH6.6 condition via two-way analysis of variance (ANOVA) with a Tukey's post-hoc test). **g, h, i** BMM secreted anti-inflammatory factor IL-10 at different pHs (n=3, values were mean ± std. dev., $p<0.0001$ when comparing MSaP-aL/p and other four groups on the 3rd, 5th and 7th day at pH5.8 condition, and $p<0.0001$ when comparing MSaP-aL/p and other four groups on the 5th and 7th day at pH6.6 condition via two-way analysis of variance (ANOVA) with a Tukey's post-hoc test). **j, k, l** BMM secreted pro-inflammatory factor TGF-β at different pHs (n=3, values were mean ± std. dev., $p<0.0001$ when comparing MSaP-aL/p and other four groups on the 3rd, 5th and 7th day at pH5.8 condition, and $p<0.0001$ when comparing MSaP-aL/p and other four groups on the 5th and 7th day at pH6.6 condition via two-way analysis of variance (ANOVA) with a Tukey's post-hoc test).

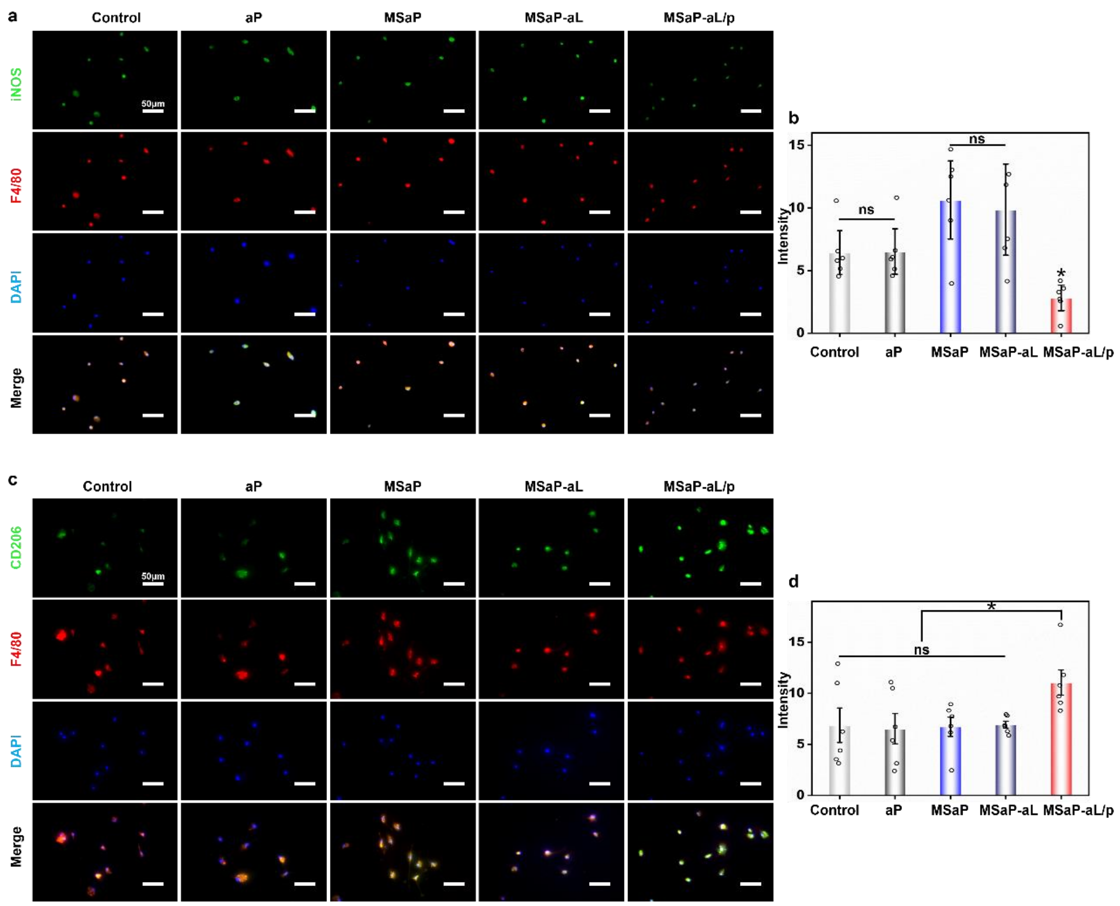

Supplementary Figure 7. Polarization of BMMs by immunofluorescence staining. **a** Immunofluorescence staining of macrophage marker F4/80 (red) and M1 macrophage marker iNOS (green) (scale bars were 50μm, 3 independent experiments). **b** Fluorescence semi-quantitative analysis of M1 macrophage marker iNOS (n=6, values were mean ± std. dev., $^{*}p < 0.05$ when comparing MSaP-aL/p and other groups via one-way analysis of variance (ANOVA) with a Tukey's post-hoc test., ns, not significant). **c** Macrophage marker F4/80 (red) and M2 macrophage marker CD206 (green) immunofluorescence staining (scale bars were 50μm, 3 independent experiments). **d** Fluorescence semi-quantitative analysis of M2 macrophage marker CD206 (n=6, values were mean ± std. dev., $^{*}p < 0.05$ when comparing MSaP-aL/p and other groups via one-way analysis of variance (ANOVA) with a Tukey's post-hoc test., ns, not significant).

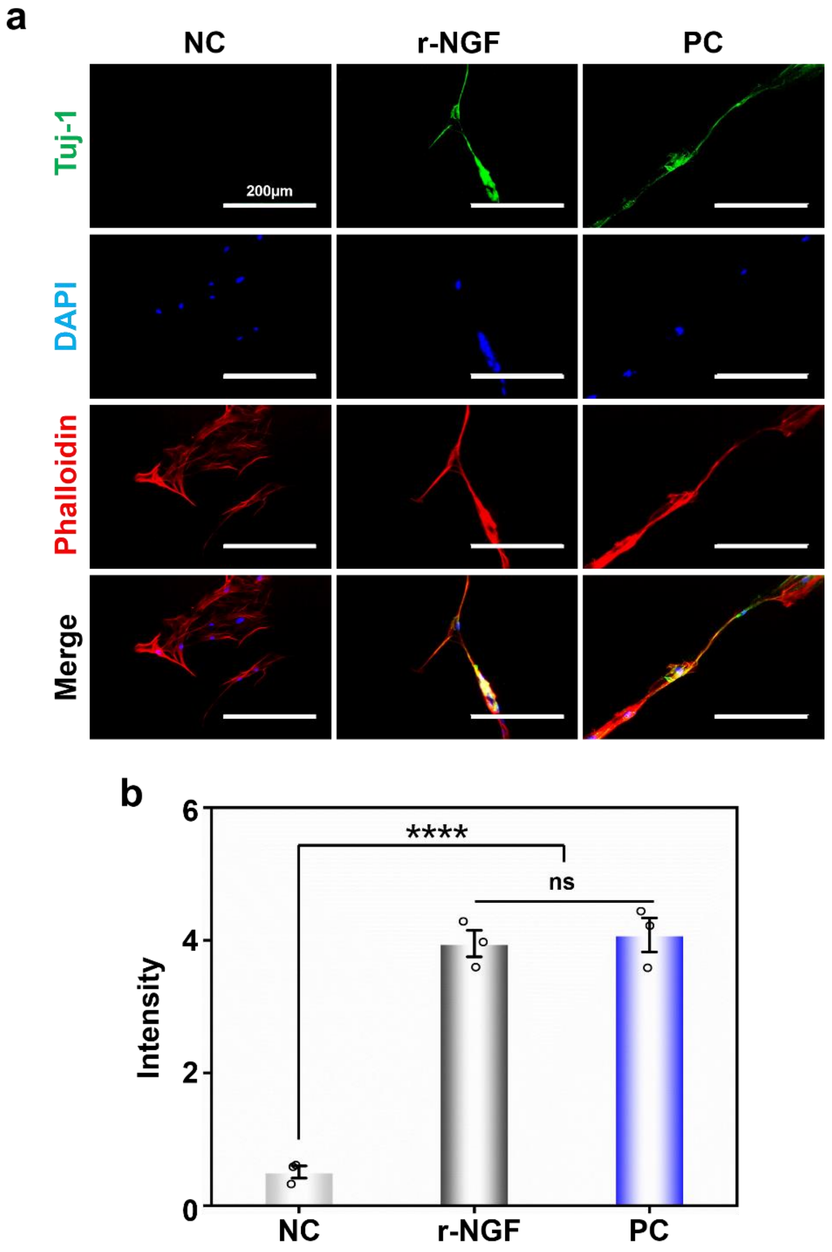

Supplementary Figure 8. Long-term biological activity of NGF. a Immunofluorescence staining of Tuj-1 staining images on cell culture plate (NC), release solution group (r-NGF) and cytokine NGF group (PC) with **b**, Corresponding fluorescence semi-quantitative analysis (n=3, all values were mean ± std. dev., $^{****}p < 0.0001$ when comparing r-NGF, PC and NC group via one-way analysis of variance (ANOVA) with a Tukey's post-hoc test., ns, not significant, scale bars were 200µm, 3 independent experiments).

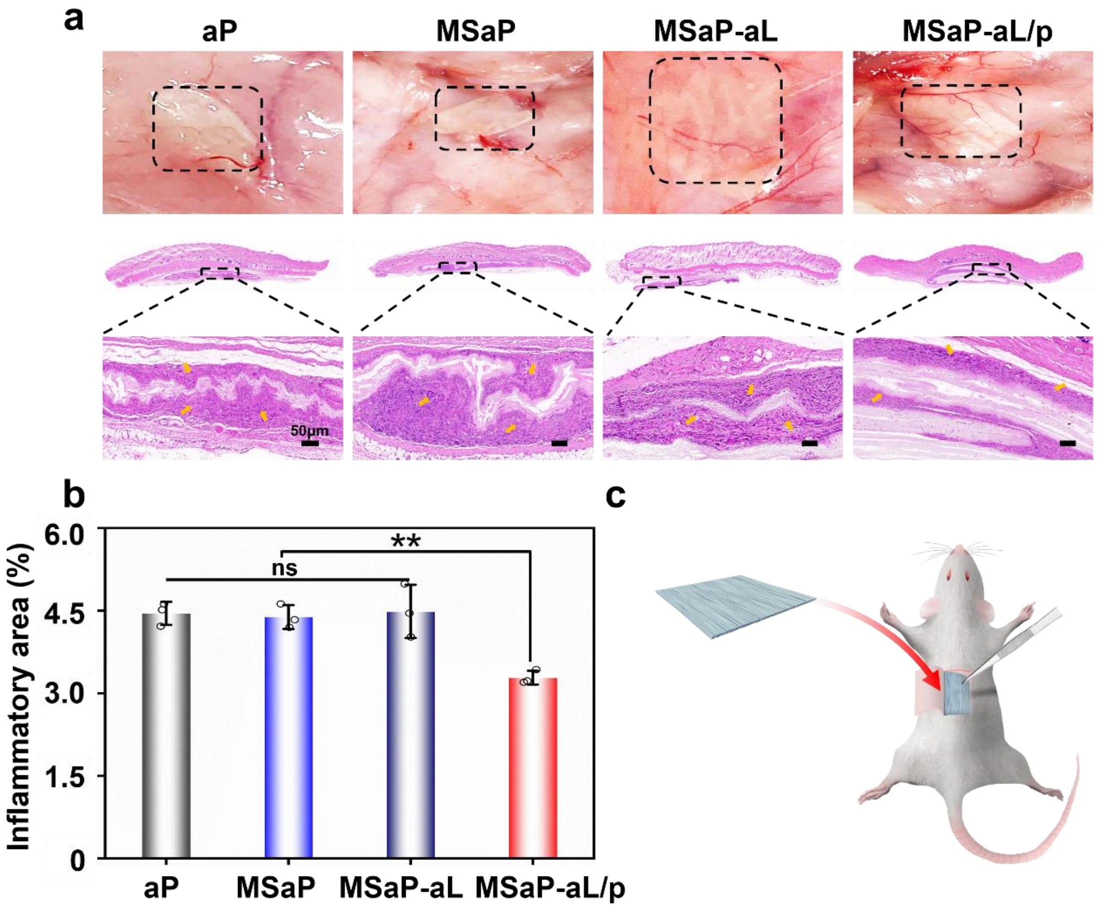

Supplementary Figure 9. Foreign body reaction of fiber scaffolds. a General observation and corresponding H&E staining after subcutaneous implantation on the back of rats for 2 weeks (scale bars were 50μm, 3 independent experiments). The yellow arrow refers to the inflammatory response area around the fiber scaffolds. **b** Analysis of inflammatory band area of HE staining by ImageJ software (n=3, all values were mean ± std. dev., $^{**}p < 0.01$ when comparing MSaP-aL/p and other groups via one-way analysis of variance (ANOVA) with a Tukey's post-hoc test., ns, not significant). **c** Schematic diagram of fibrous scaffolds implanted subcutaneously on the back of rats.

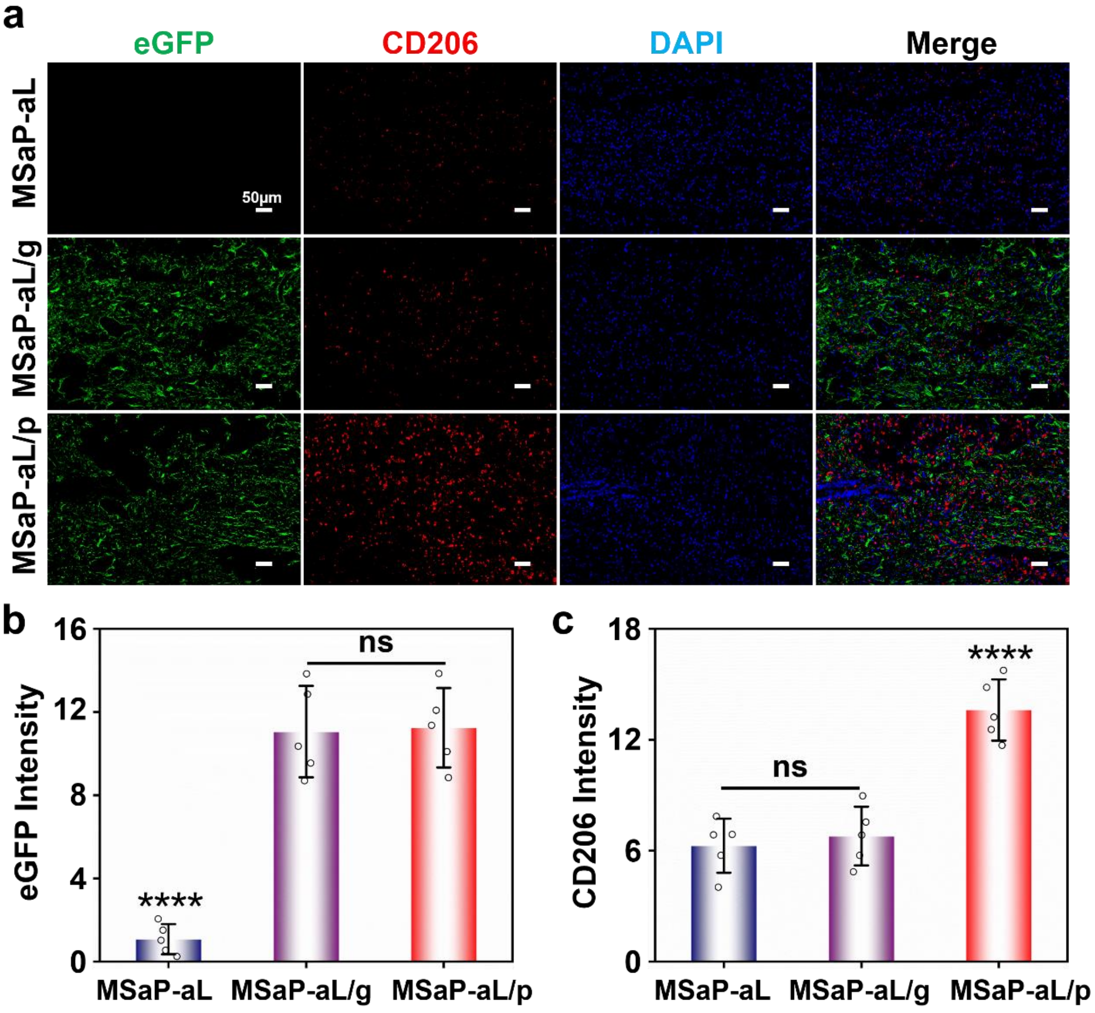

Supplementary Figure 10. IL-4-eGFP transfection (green) and staining of M2 macrophages (red) of spinal cord tissues in MSaP-aL, MSaP-aL/g and MSaP-aL/p at Day 7. a EGFP expression and CD206 immunofluorescence staining in each group (scale bars are 50μm, 4 independent experiments). **b** Semi-quantitatively analysis of eGFP (n=5, values were mean ± std. dev., $^{****}p < 0.0001$ when comparing MSaP-aL and other groups via one-way analysis of variance (ANOVA) with a Tukey's post-hoc test., ns, not significant). **c** Semi-quantitatively analysis of CD206 (n=5, all values were mean ± std. dev., $^{****}p < 0.0001$ when comparing MSaP-aL/p and other groups via one-way analysis of variance (ANOVA) with a Tukey's post-hoc test., ns, not significant).

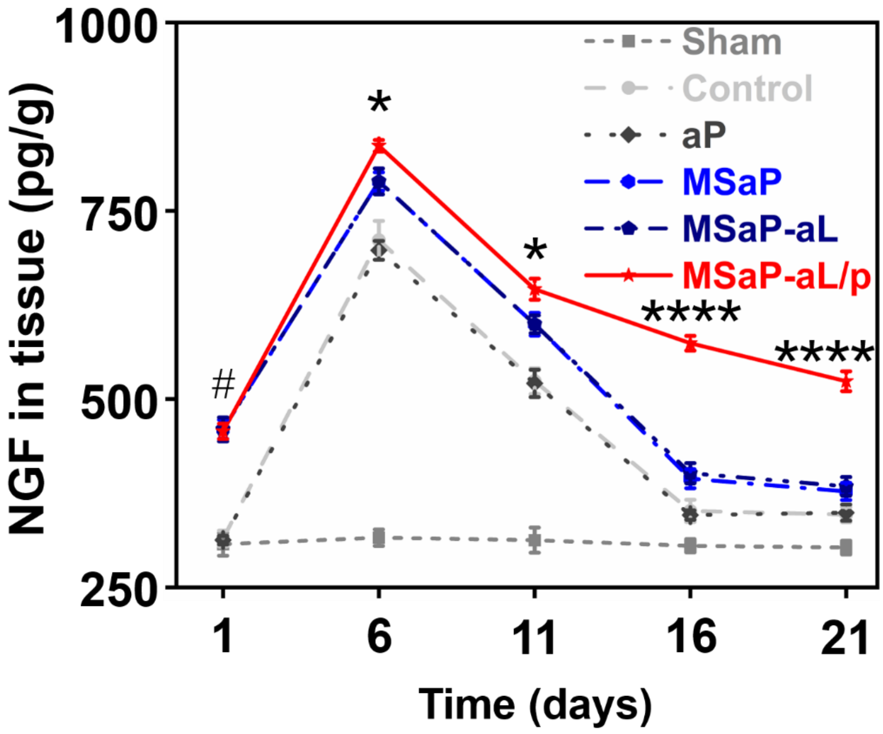

Supplementary Figure 11. Pharmacokinetics of NGF in spinal cord after operation (n=3, all values were mean ± std. dev., $^{\#}p < 0.0001$ when comparing microsol fiber groups and other groups, $^{*}p < 0.05$, $^{****}p < 0.0001$ when comparing MSaP-aL/p and other groups via one-way analysis of variance (ANOVA) with a Tukey's post-hoc test)

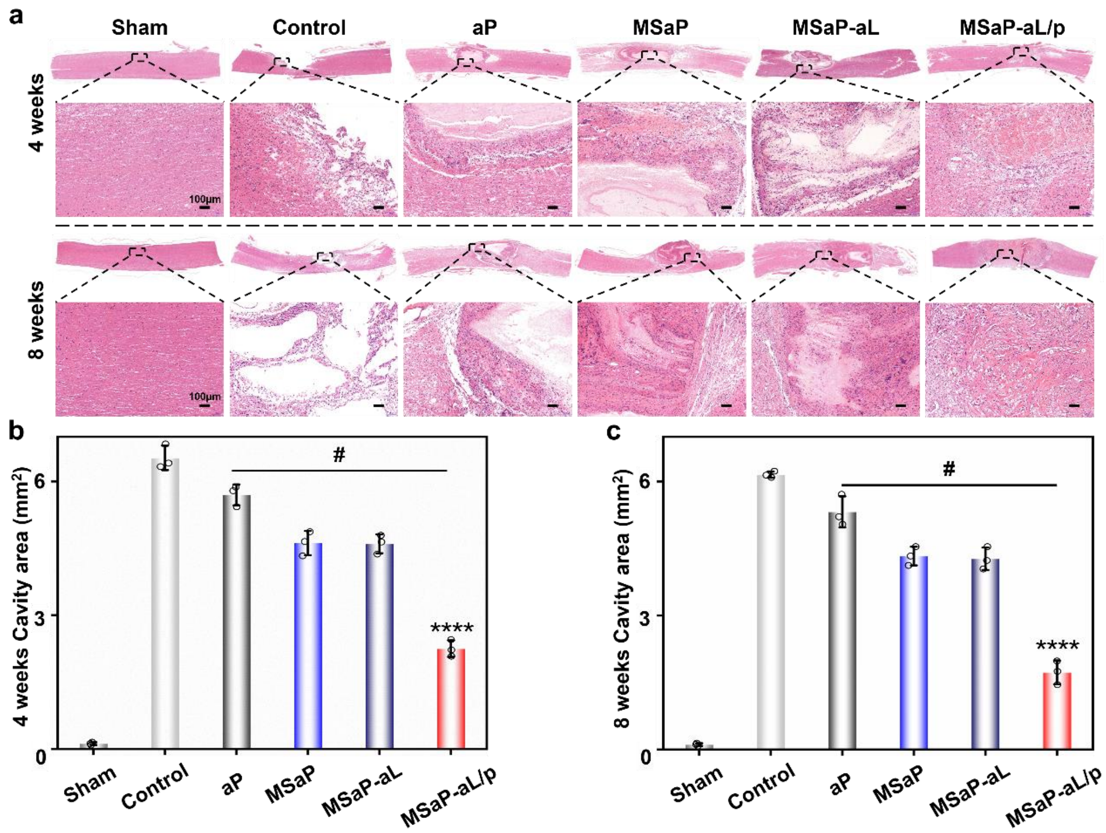

Supplementary Figure 12. Spinal cord specimens were stained with H&E. a H&E staining of rat spinal cord at 4w and 8w, respectively (scale bars were 100μm, 4 independent experiments). **b** Calculation of spinal cavity area at 4 weeks after surgery (n=3, values were mean ± std. dev., $^{****}p < 0.0001$ when comparing MSaP-aL/p and other groups; $^{\#}p < 0.001$ when comparing fiber bundle implantation groups (aP, MSaP, MSaP-aL, MSaP-aL/p) and control group via two-way analysis of variance (ANOVA) with a Tukey's post-hoc test). **c** Calculation of spinal cavity area at 8 weeks after surgery (n=3, values were mean ± std. dev., $^{****}p < 0.0001$ when comparing MSaP-aL/p and other groups; $^{\#}p < 0.001$ when comparing fiber bundle implantation groups (aP, MSaP, MSaP-aL, MSaP-aL/p) and control group via two-way analysis of variance (ANOVA) with a Tukey's post-hoc test).

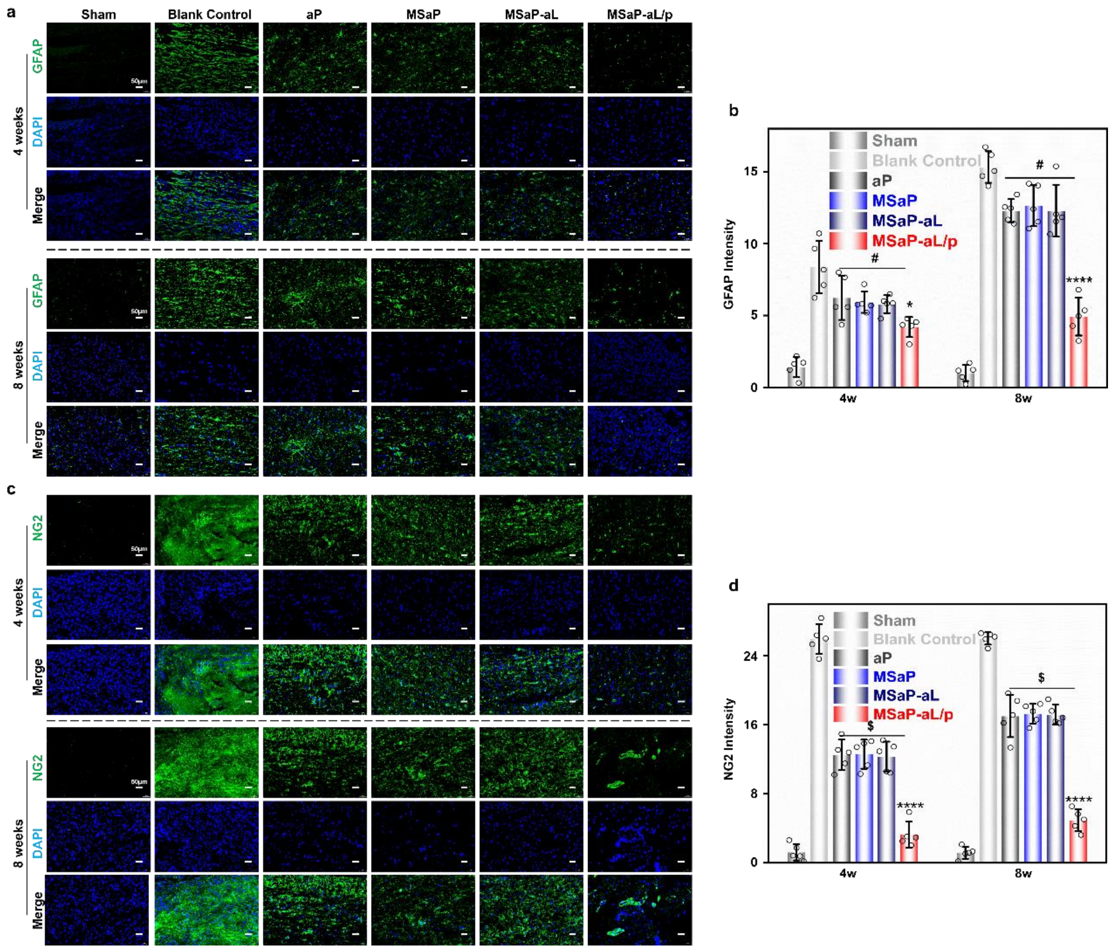

Supplementary Figure 13. Immunofluorescence staining of activated astrocytes and glial scar. a, c Immunofluorescence staining of astrocytes (scale bars were 50μm, 3 independent experiments) and quantitatively analyzed with optical density along with **b, d** their quantification with optical density (n=5, all values were mean ± std. dev., $^{*}p < 0.05$, $^{****}p<0.0001$ when comparing MSaP-aL/p and other control groups at the same time point. $^{\#}p<0.05$ when comparing material transplantation groups and blank control group via two-way analysis of variance (ANOVA) with a Tukey's post-hoc test at the same time point of GFAP staining. $^{\$}p<0.0001$ when comparing material transplantation groups and blank control group via two-way analysis of variance (ANOVA) with a Tukey's post-hoc test at the same time point of NG2 staining).

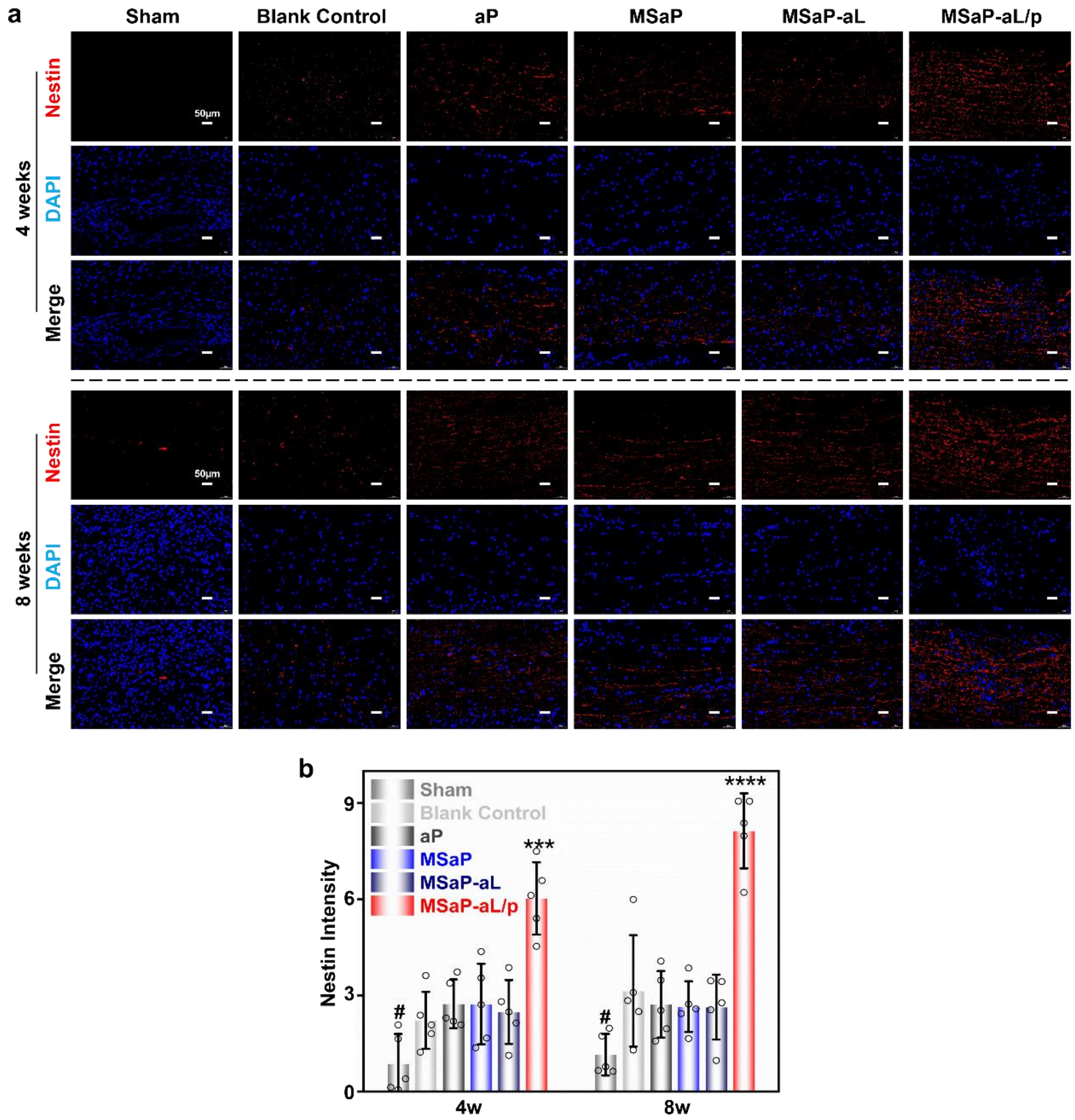

Supplementary Figure 14. Immunofluorescence staining of neural stem cells and neuron. a, Immunofluorescence staining of neural stem cells (scale bars were 50μm, 3 independent experiments) with **b** its quantification analyzed with optical density (n=5, Nestin intensity values were mean ± std. dev., $^{\#}p<0.05$ when comparing sham group and other groups and $^{***}p<0.001$, $^{****}p<0.0001$ when comparing MSaP-aL/p and other control groups via two-way analysis of variance (ANOVA) with a Tukey's post-hoc test at the same time point).

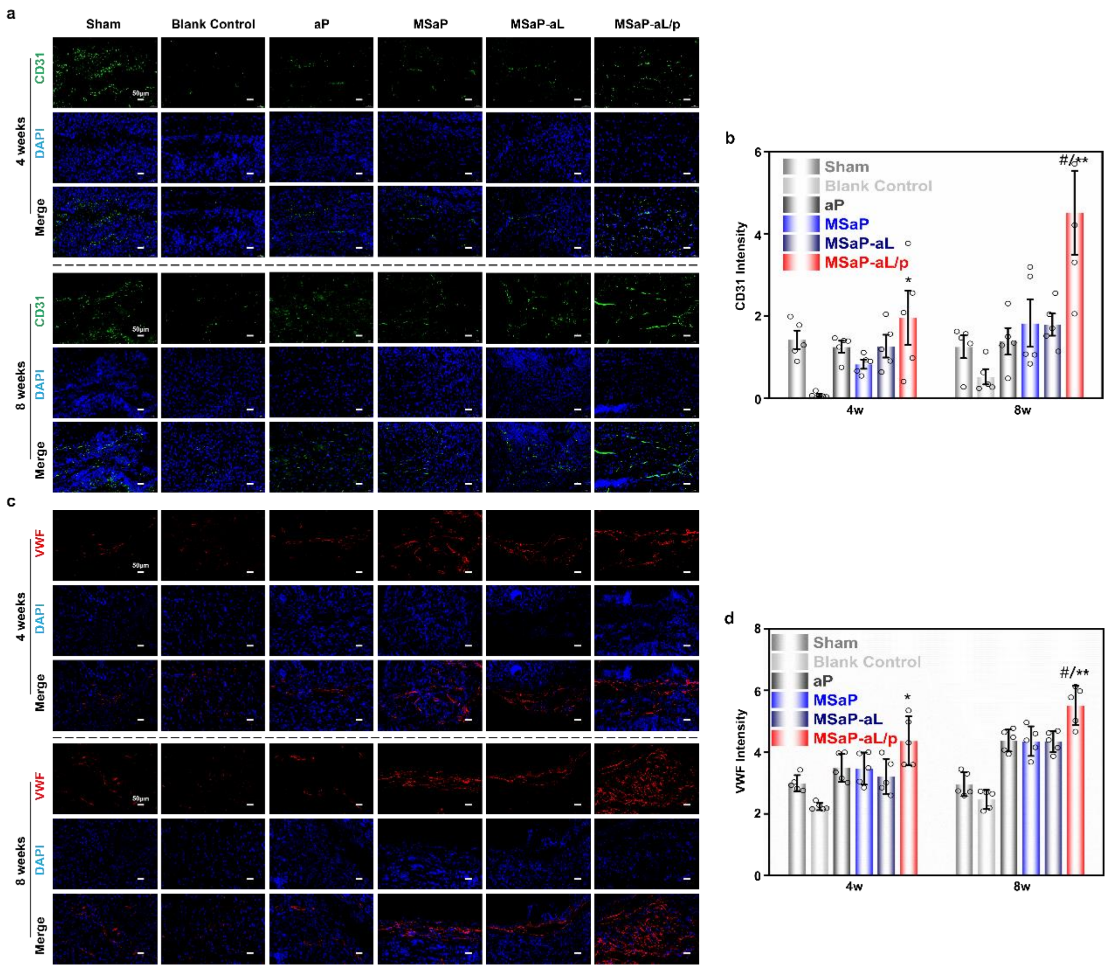

Supplementary Figure 15. Vascularization ability of different fiber bundles. a, Immunofluorescence staining of vascular endothelial cells (scale bars were 50μm, 4 independent experiments) and **b** their quantitatively analysis with optical density. **c,** Immunofluorescence staining of neovascularization (scale bars were 50μm, 3 independent experiments) and **d** their quantitatively analysis with optical density (n=5, CD31 and VWF values were mean ± std. dev., $^{*}p<0.05$, $^{**}p<0.01$ when comparing MSaP-aL/p and other control groups at the same time and $^{\#}p<0.05$ when comparing 4w and 8w in MSaP-aL/p via two-way analysis of variance (ANOVA) with a Tukey's post-hoc test).

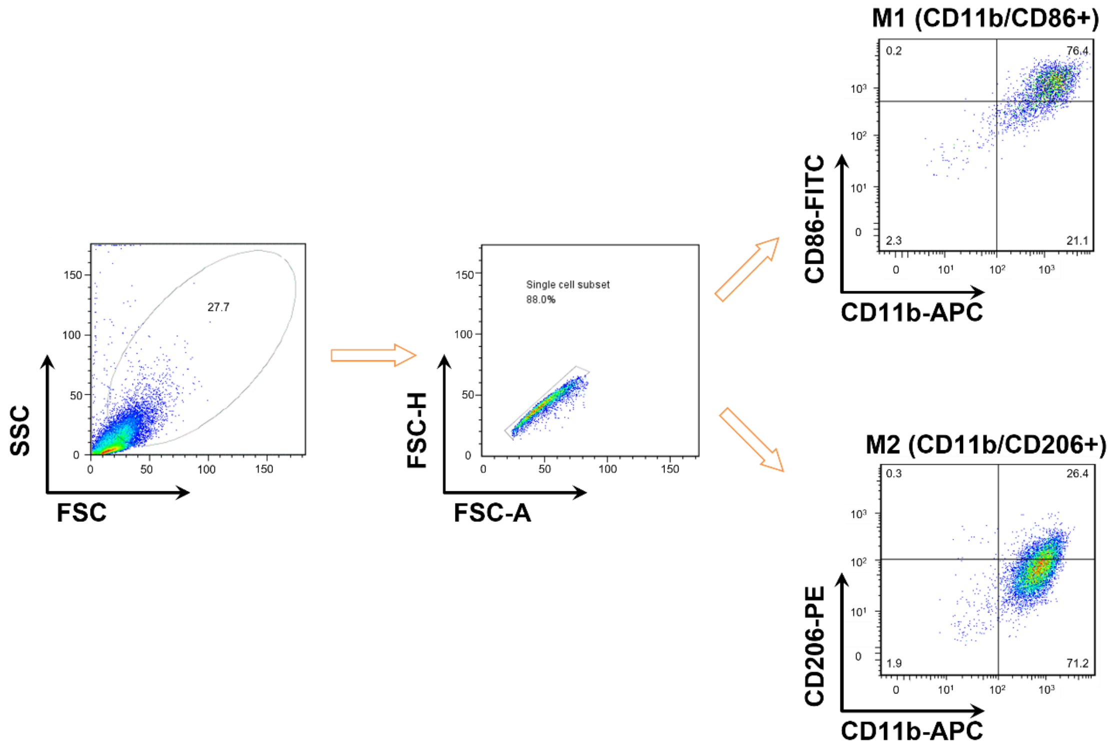

Supplementary Figure 16. Sequential gating of Fig. 9a

Supplementary Tables

Supplementary Table 1. Material grouping.

Group	Denoted	Function
PLA electrospinning	PLA	Control
Amino PLA electrospinning	aP	Control
Amino PLA microsol electrospinning	MSaP	Control
Amino PLA microsol electrospinning carrying blank cationic liposomes	MSaP-L	Control
Amino PLA microsol electrospinning carrying blank aldehyde cationic liposomes	MSaP-aL	Control
Amino PLA microsol electrospinning carrying aldehyde cationic liposome loading eGFP plasmid	MSaP-aL/g	Control
Amino PLA microsol electrospinning carrying aldehyde cationic liposomes loading IL-4 plasmid	MSaP-aL/p	Test

Supplementary Table 2. Sample grouping.

	1:1	1:1.5	1:2	1:2.5	1:3
pDNA	8μg	8μg	8μg	8μg	8μg
aLiposome	8μl	12μl	16μl	20μl	24μl

Supplementary Table 3. Configure PBS for different pH.

pH	0.2M Na_2HPO_4（ml）	0.2M NaH_2PO_4（ml）
7.4	19	81
6.6	62.5	37.5
5.8	92	8

Supplementary Table 4. qRT-PCR immune factor primers in vitro.

Gene	Primer	Sequence	Tm (℃)
IL-1β	Forward	GAAAGACGGCACACCCACC	62
	Reverse	AAACCGCTTTTCCATCTTCTTCT	54.86
TNF-α	Forward	TACTGAACTTCGGGGTGATTGGTCC	62.22
	Reverse	CAGCCTTGTCCCTTGAAGAGAACC	62.11
IL-10	Forward	GGTTGCCAAGCCTTGTCAGAA	57.80
	Reverse	GCTCCACTGCCTTGCTTTTATT	56.26
TGF-β	Forward	CTTCAGCTCCACAGAGAAGAACTGC	62.22
	Reverse	CACGATCATGTTGGACAACTGCTCC	62.22
GAPDH	Forward	AACTCCCATTCTTCCACCT	56
	Reverse	TTGTCATACCAGGAAATGAGC	53.90

Supplementary Table 5. qRT-PCR nerve specific marker primers.

Gene	Primer	Sequence	Tm (℃)
Tuj-1	Forward	TATCTTCGGTCAGAGTGGTG	60
	Reverse	CATCCAGGACTGAGTCCAC	60
NSE	Forward	CGGAACTATCCTGTGGTCTC	62
	Reverse	GACATTGGCTGTGAACTTGG	62
Tau	Forward	ACGATTTCTGCTCCATGGTC	60
	Reverse	AAGGTGACCTCCAAGTGTGG	60
NF-200	Forward	GTTCCGAGTGAGATTGGAC	58
	Reverse	GTTATCTCCTCTTGGGCAG	58
GAPDH	Forward	AACTCCCATTCTTCCACCT	56
	Reverse	TTGTCATACCAGGAAATGAGC	53.9

Supplementary Table 6. The primer sequence of immune factor for qRT-PCR in vivo.

Gene	Primer	Sequence	Tm (°C)
IL-4	Forward	GCAACAAGGAACACCACGG	60
	Reverse	AAGCACGGAGGTACATCACGT	57.8
TNF-α	Forward	TACTGAACTTCGGGGTGATTGGTCC	62.22
	Reverse	CAGCCTTGTCCCTTGAAGAGAACC	62.11
IL-1β	Forward	GAAAGACGGCACACCCACC	62
	Reverse	AAACCGCTTTTCCATCTTCTTCT	54.86
IL-10	Forward	CCCTCTGGATACAGCTGCG	62
	Reverse	GCTCCACTGCCTTGCTTTTATT	56.26
TGF-β	Forward	CTTCAGCTCCACAGAGAAGAACTGC	62.22
	Reverse	CACGATCATGTTGGACAACTGCTCC	62.22
GAPDH	Forward	AACTCCCATTCTTCCACCT	56
	Reverse	TTGTCATACCAGGAAATGAGC	53.90

Supplementary Reference

1. Cossich, E. et al., Development of electrospun photocatalytic TiO2-polyamide-12 nanocomposites. *Biotechnol. Adv.* **31**, 421-437 (2015).

2. McMahon, S. S. et al., Engraftment, migration and differentiation of neural stem cells in the rat spinal cord following contusion injury. *Cytotherapy.* **12**, 313-325 (2010).
